# Allergen bronchoprovocation: correlation between FEV_1_ maximal percent fall and area under the FEV_1_ curve and impact of allergen on recovery

**DOI:** 10.1186/s13223-023-00759-6

**Published:** 2023-01-21

**Authors:** Sarah-Marie Durr, Beth Davis, Gail Gauvreau, Donald Cockcroft

**Affiliations:** 1 Department of Medicine, University of Saskatchewan, Saskatoon, SK Canada; 2 Department of Medicine, McMaster University, Hamilton, ON Canada; 3 Royal University Hospital, 103 Hospital Drive, Saskatoon, SK S7N0W8 Canada

**Keywords:** Asthma, Allergens, Allergen bronchoprovocation test, FEV_1_, Area under the curve

## Abstract

**Background:**

House dust mite (HDM) induces greater responses than other allergens during allergen bronchoprovocation (ABP) testing. The two standardized methods for reporting results of ABP tests are the maximal percent fall in forced expiratory volume in one second (FEV_1, max_; %) and the area under the FEV_1_ vs time curve (AUC; %FEV_1_ x min). The relationship between these methods has not been previously investigated.

**Aims:**

We aimed to measure the correlation between FEV_1, max_ and AUC during the early asthmatic response (EAR) and the late asthmatic response (LAR), and to determine if the EAR recovery period for HDM would be longer than other allergens (cat, grass, horse, and ragweed).

**Methods:**

We retrospectively calculated the AUC and correlation between FEV_1, max_ and AUC during the EAR_(0-2 h)_ and LAR_(3-7 h)_ for each allergen. We compared EAR_(0-3 h)_ and LAR_(3-7 h)_ FEV_1, max_, AUC and absolute difference in FEV_1, max_ to the most recovered FEV_1_ (FEV_1, min_). We performed pairwise comparisons of correlation and slope values using Fischer’s r to z transformation and t-tests, respectively. AUC and absolute differences in FEV_1, max_ and FEV_1, min_ were compared using a one-way ANOVA test, followed by a *post-hoc* Scheffe test.

**Results:**

Correlation between the FEV_1, max_ and AUC during the EAR_(0-2 h)_ (n = 221) was 0.807, and was 0.798 during the LAR_(3-7 h)_ (n = 157 of 221), (difference p = 0.408). The EAR_(0-3 h)_ AUC and FEV_1, max_ did differ between allergens (both p < 0.0001) but the LAR_(3-7 h)_ AUC and FEV_1, max_ did not (p = 0.548 and 0.824, respectively). HDM did not have a larger AUC or FEV_1, max_, than all other allergens during the EAR_(0-3 h)_ or the LAR_(3-7 h)_. The absolute difference between the FEV_1, max_ and FEV_1, min_ during the EAR_(0-3 h)_ did not differ between allergens (p = 0.180).

**Conclusion:**

The FEV_1, max_ and AUC for both the EAR_(0-2 h)_ and LAR_(3-7 h)_ had excellent correlation, with no significant difference. Thus, significant bronchoconstriction will likely result in a longer recovery period. There was no evidence of delayed EAR_(0-3 h)_ recovery following HDM challenges, so HDM did not induce a larger response compared to all the other allergens examined.

Registration: Not registered. This is not a clinical trial. (This study is a retrospective analysis of data collected during several registered trials.)

## Introduction

The allergen bronchoprovocation (ABP) test is used to study asthma pathophysiology and pharmacological agents and is performed by administering serial concentrations of a relevant allergen to induce bronchoconstriction. The early asthmatic response (EAR) is defined as a ≥ 20% decrease in forced expiratory volume in one second (FEV_1_) and will usually resolve without treatment within 2 h [1, 2]. Bronchoconstriction during the EAR is usually greatest within 20 min post-challenge [3]. Late asthmatic responses (LARs), which are characterized by bronchoconstriction defined as a ≥ 15% decrease in FEV_1_ during the 3 to 7 h (or longer) timeframe post-challenge are reported to manifest in as few as 34.5% [3], and as many as 50% of early responders which may depend on geographical location [1, 2].

There are two units of measure used to quantify ABP results: the maximal percent fall in FEV_1_ (FEV_1, max_) and the area under the FEV_1_ versus time curve (AUC). While the FEV_1, max_ provides insight on the severity of bronchoconstriction, the AUC includes the duration of the response. Generally, AUC is preferred over FEV_1, max_ because it is less sensitive to outliers [2]. Nonetheless, both methods are considered reproducible and sensitive [4, 5]. A relationship must exist between the FEV_1, max_ and AUC values, but their correlation has not been previously investigated. Understanding the relationship of the two units of measure will help guide future research when deciding which, if not both, measure can be used. Pharmacological studies utilizing ABP tests are often most interested in the recovery, and the FEV_1, max_ and AUC are both used to represent recovery in different ways. The correlation between FEV_1, max_ and AUC would indicate how well these two interpretations of recovery are related: whether the degree of bronchoconstriction reflects the duration of the response. Calculating both the FEV_1, max_ and AUC would be valuable if the values were not well linked. However, if the valued had a strong association, this would mean one value could help predict the other. In addition, the longer time period of the LAR could allow for a greater variation in AUC since it is dependent on the length of the response, while the FEV_1, max_ only depends on the time point with the greatest degree of bronchoconstriction. Thus, a strong correlation between the two measures would also show that the AUC is a useful measure even during longer recovery periods like the LAR.

In addition, the choice of allergen administered during the ABP test can impact the response. House dust mite (HDM) allergen has been shown to cause greater ABP responses. Previous research has found that HDM caused a larger FEV_1, max_ during the LAR when compared to pollen challenges with EARs of similar magnitude [6]. Furthermore, HDM caused greater FEV_1, max_ at every time point compared to cat allergen [7]. All allergens activate the immunoglobulin-E (IgE) pathway to cause mast cell and basophil degranulation resulting in bronchoconstriction [8]. However, HDM may cause more severe bronchoconstriction by activating additional proteolytic pathways [9]. A major factor in bringing about an immune response to HDM allergen may be Der p 1, which has shown cysteine protease activity; it was found to cleave proteins on the IL-2 receptor [10, 11], leading to a possible immune bias for T_H_2 cells, increasing allergic hypersensitivity [9]. Chronic exposure to HDM may also increase responsiveness to an ABP challenge, contributing to greater responses. [6]. Determining if HDM is associated with a longer recovery period after the EAR would provide further evidence that this allergen causes more severe outcomes in ABP tests and help support the current understanding of the excessive bronchoconstriction compared to other allergens. The existing research on the response to HDM allergen is focused on the FEV_1, max_; we expanded on this understanding by also measuring the AUC. We also included a larger number of both seasonal and perennial allergens (cat, grass, HDM, horse, and ragweed) to compare the responses to a greater variety of allergens. The focus on recovery after an HDM challenge is also clinically relevant, as it will help put the theoretical understanding of the additional proteolytic pathway HDM may activate, in terms of measures of recovery.

One of our objectives was to understand how strong the relationship is between FEV_1, max_ and AUC during the EAR_(0-2 h)_ as well as during the LAR_(3-7 h)_. We hypothesized that the FEV_1, max_ and AUC should have good to excellent correlation (r ≥ 0.8) for both the EAR_(0-2 h)_ and the LAR_(3-7 h)_; but, we suspected it would be greater for the EAR_(0-2 h)_ based on the nature of the ABP test, with allergen administration stopping once a 20% fall in FEV_1_ is reached. Understanding the correlation between these endpoints is important to ABP tests: it will allow us to determine how the magnitude or duration of the response is linked to FEV_1, max_. We also set out to determine if the EAR recovery after an HDM challenge would be significantly longer than that of other allergens.

## Methods

We retrospectively gathered ABP data from our Clinical Investigator Collaborative database using data from the University of Saskatchewan and McMaster University. Participants included non-smokers aged 18 to 63 who took part in studies conducted from 2002 to 2019. We looked at screening allergen challenges which do not include any treatment intervention. The allergens examined, namely, cat, grass, HDM, horse, and ragweed, were selected based on the study participant’s clinical history and allergen prick skin test results [1, 2]. Participants who took part in more than one study had their data included only once, using the most recent data. ABP tests were performed as previously described [1, 2]. Participants inhaled allergens selected based on their skin prick test results in conjunction with their own reporting of allergy symptoms. FEV_1_ was measured 10 min, 20 min, 30 min, 45 min, 60 min, 90 min, 2 h, 3 h, 4 h, 5 h, 6 h, and 7 h post-challenge.

The EAR_(0-2 h)_ FEV_1, max_ was the largest percent fall in FEV_1_ relative to baseline (i.e., pre-allergen inhalation) from 0 to 2 h post-challenge, and the LAR_(3-7 h)_ FEV_1, max_ was the largest percent fall in FEV_1_ from 3 to 7 h post-challenge; each being at least 20% and 15% respectively. If a single time point during the ABP test was missing, it was estimated using a weighted average of the preceding and following percent fall in FEV_1_ values. AUC for the EAR_(0-2 h)_ and LAR_(3-7 h)_ were calculated from percent fall in FEV_1_ versus time data using the trapezoid rule. The absolute difference in FEV_1, max_ and the most recovered (i.e., least) percent fall in FEV_1_ (FEV_1, min_) that followed during EAR_(0-3 h)_ was calculated to determine the magnitude of recovery.

Scatterplot graphical representations for correlation analyses were constructed for all allergens combined and for individual allergens (cat, grass, HDM, horse, and ragweed) both for the EAR_(0-2 h)_ and LAR_(3-7 h)_. Pearson’s correlation coefficient and the slope of the regression line were calculated using Microsoft Excel (Version 16.60). Pairwise comparisons of correlation were done using Fischer’s r to z transformation [12]. Pairwise comparisons of slopes were done using a t-test [12]. AUC and absolute differences in FEV_1, max_ and FEV_1, min_ were compared using a one-way ANOVA test. A significant ANOVA test was followed by a *post-hoc* Scheffe test. Significance at the 5% level was tested.

We believed that a slower recovery period after an HDM challenge would manifest as a larger EAR_(0-3 h)_ AUC; the third hour was included to ensure we were measuring recovery at the last stage of the EAR, allowing us to see if any participants still had significant bronchoconstriction. Determining if HDM is associated with a longer recovery period after the EAR would provide further evidence that this allergen causes more severe outcomes in ABP tests and help support the current understanding of the excessive bronchoconstriction compared to other allergens.

## Results

### Participant characteristics

Data from 221 participants were used for EAR_(0-2 h)_ analysis, 157 of the 221 participants (71%) were dual responders, defined as a ≥ 15% decrease in FEV_1_ during the 3 to 7 h post-challenge, and these data were used for LAR_(3-7 h)_ analyses. Three participants had a single time point missing during the ABP test (one each at 20 min, 30 min, and 45 min). The most common allergen used for ABP testing was HDM followed by cat, grass, ragweed, and horse (Table 1).

**Table 1:** Participant characteristics during the EAR and LAR

	EAR	LAR
n	221	157
Sex (F: M)	129: 92	96: 61
Age (years)	27 ± 9	27 ± 10
Height (m)	1.66 ± 0.27	1.68 ± 0.21
Baseline FEV_1_ (L)	3.47 ± 0.74	3.45 ± 0.69
Baseline FEV_1_ (% predicted)	91.9 ± 12.2	92.4 ± 12.8
Race		
African America	4	3
American Indigenous	1	1
Asian	15	9
South Asian	4	2
Hispanic	1	0
Middle Eastern	1	1
White Caucasian	141	97
Caucasian/Asian	1	1
Not Recorded	53	43
Allergen		
Alternaria	5	5
Cat	64	40
Grass	32	21
HDM	72	58
Horse	20	8
Ragweed	26	24
Tree	2	1

Results are represented as means ± standard deviations (SD). F, female; M, male

### Overview of EAR and LAR responses

**Fig. 1:**
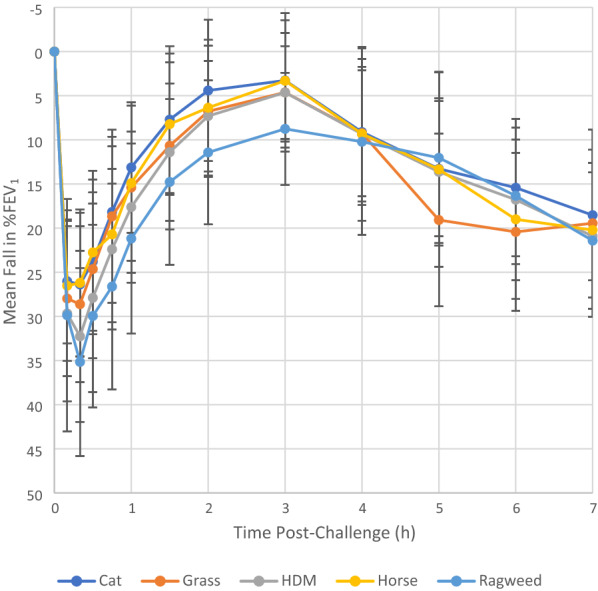
Mean fall in FEV_1_ (%) and standard deviation for common allergens (cat, grass, HDM, horse, and ragweed)

Mean fall in FEV_1_ at each timepoint post-inhalation for each allergen are shown in Fig. 1. During the whole 7-h post-challenge timeframe, ragweed inhalation generated the largest fall in FEV_1_ (36.9%) followed by HDM (35.5%), grass (31.5%), cat (30%) and horse (29.7%). Recovery from ragweed inhalation required the most time (i.e., largest EAR_(0-3 h)_ AUC) and is the least complete (8.8% bronchoconstriction remains at 3 h post-challenge). Cat and horse exhibit the least bronchoconstriction and recover more quickly (i.e., small EAR_(0-3 h)_ AUCs) and more completely (i.e., FEV_1_ returns to within 5% of baseline at 3 h post-challenge). LAR responses are relatively similar across the 3–7-h post-challenge timeframe in terms of both maximal fall in FEV_1_ and AUC for cat, horse and HDM; these responses are developing gradually hour by hour. Late responses to grass develop rapidly between 4 and 6 h and beginning to recover at 7 h. The FEV_1_ decrease during the LAR to ragweed is initially slight (approximately 3% over hours 4 and 5) then nearly doubles over the next two hours with a steep downward trend and no evidence of recovery at 7 h post-challenge.

### *FEV*_*1, max*_* vs AUC Correlation and Slope*

**Fig. 2:**
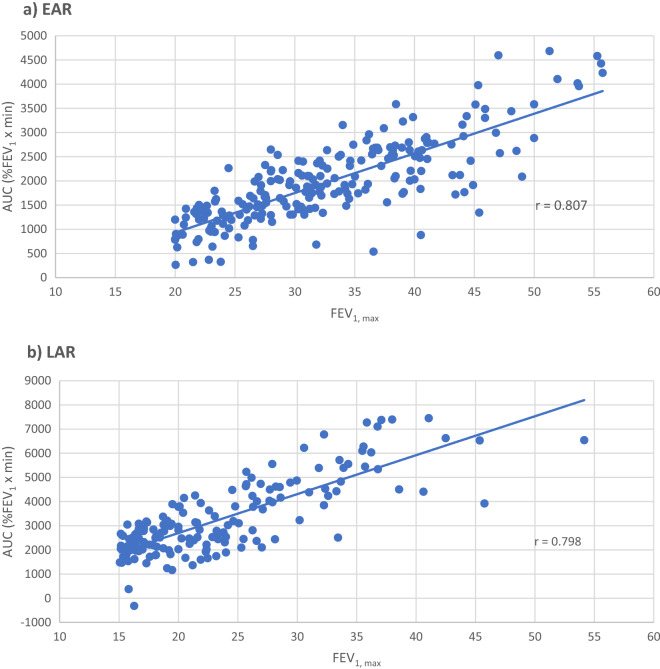
FEV_1, max_ vs AUC for all allergens combined (cat, grass, HDM, horse, ragweed, *Alternaria*, and tree) during the **a** EAR_(0-2 h)_ and **b** LAR_(3-7 h)_

The correlation of FEV_1, max_ vs AUC for all allergens combined during the EAR_(0-2 h)_ (n = 221) was 0.807 (Fig. 2a), and during the LAR_(3-7 h)_ (n = 157 of 221 i.e., dual responders) was 0.798 (Fig. 2b); these correlation values did not differ statistically (p = 0.408). The points in Fig. 2a become more dispersed after FEV_1, max_ values of 45, and some outliers exist where the FEV_1, max_ values (approximately 30–45) have relatively small AUC values. Points become more dispersed in Fig. 2b after FEV_1, max_ values of 35, with some outliers in the FEV_1, max_ range of 45–55 with relatively small AUCs.

**Fig. 3:**
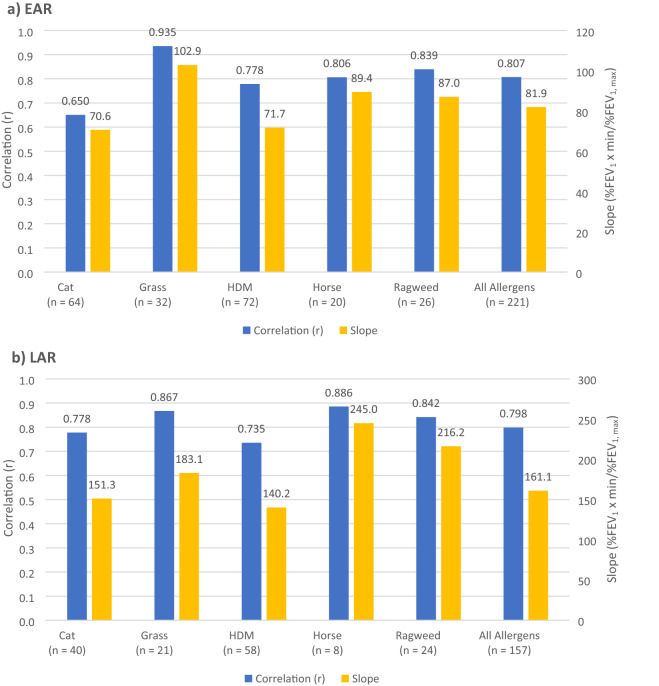
FEV_1, max_ vs AUC Correlation and slope values for individual allergens (cat, grass, HDM, horse, and ragweed) and all allergens combined (cat, grass, HDM, horse, ragweed, *Alternaria*, and tree) during the **a** EAR_(0-2 h)_ and **b** LAR_(3-7 h)_

The slope (units %FEV_1_ x min/%FEV_1, max_) of the regression lines for the EAR_(0-2 h)_ (Fig. 3a) and LAR_(3-7 h)_ of all allergens (Fig. 3b) differed statistically (81.9 and 161.1, respectively; p < 0.0001). During the EAR_(0-2 h)_, the relationship between AUC and FEV_1, max_ for cat, grass, HDM, horse, and ragweed individually were 0.650, 0.935, 0.778, 0.806, and 0.839, respectively. The significant pairwise comparisons for EAR_(0-2 h)_ correlations were of grass vs cat (p < 0.0001), grass vs HDM (p = 0.001), grass vs horse (p = 0.028), and grass vs ragweed (p = 0.042). Cat vs ragweed was also significantly different (p = 0.035), with ragweed having the greater correlation. All other EAR_(0-2 h)_ correlation pairwise comparisons did not differ statistically (p = 0.066 to 0.391) (Table 2). The slope of the regression line during the EAR_(0-2 h)_ for each allergen (cat: 70.6, grass: 102.9, HDM: 71.7, horse: 89.4, and ragweed: 87.0) only differed statistically for grass vs cat, and grass vs HDM (p = 0.013 and 0.002, respectively) (Table 2). The correlation values for individual allergens during the LAR_(3-7 h)_ (cat: 0.778, grass: 0.867, HDM: 0.735, horse: 0.886, and ragweed: 0.842) did not have any significant pairwise comparison difference (p = 0.081 to 0.437). The slope of the regression lines for individual allergens during the LAR_(3-7 h)_ (cat: 151.3, grass: 183.1, HDM: 140.2, horse: 245.0, and ragweed: 216.2) only had one statistically different pairwise comparison of HDM to ragweed (p = 0.034) (Table 2).

**Table 2:** Correlation (r) and slope p-values for pairwise comparisons

		Cat vs Grass	Cat vs HDM	Cat vs Horse	Cat vs Ragweed	Grass vs HDM	Grass vs Horse	Grass vs Ragweed	HDM vs Horse	HDM vs Ragweed	Horse vs Ragweed
*EAR*_*(0-2 h)*_	*r*	< 0.0001	0.066	0.108	0.035	0.001	0.028	0.042	0.391	0.23	0.374
	*slope*	0.013	0.934	0.299	0.295	0.002	0.413	0.246	0.275	0.256	0.897
*LAR*_*(3-7 h)*_	*r*	0.164	0.321	0.224	0.247	0.081	0.437	0.384	0.162	0.132	0.363
	*slope*	0.313	0.674	0.102	0.073	0.152	0.294	0.39	0.062	0.034	0.637
*EAR*_*(0-2 h)*_* vs LAR*_*(3-7 h)*_											
* All Allergens*	*r*	0.408									
	*slope*	< 0.0001									

### *EAR*_*(0-3 h)*_* and LAR*_*(3-7 h)*_* AUC and Absolute Difference in FEV*_*1, max*_* and FEV*_*1, min*_

Significant differences in EAR_(0-3 h)_ AUC (units %FEV_1_ x min) between common allergens (cat: 1917.0, grass: 2264.0, HDM: 2510.2, horse: 2089.9, and ragweed: 3120.8) were identified (ANOVA p < 0.0001), with the *post-hoc* Scheffe test showing cat vs HDM, cat vs ragweed, and horse vs ragweed being statistically different. The absolute difference in FEV_1, max_ and FEV_1, min_ during the EAR_(0-3 h)_ (cat: 28.9, grass: 28.9, HDM: 31.8, horse: 28.0, and ragweed: 30.3) did not differ between allergens (ANOVA p = 0.180).

The LAR_(3-7 h)_ AUC did not differ between allergens (cat: 3003.5, grass: 3697.0, HDM: 3164.9, horse: 3325.4, and ragweed: 3238.9; ANOVA p = 0.548). Similarly, the FEV_1, max_ during the LAR_(3-7 h)_ were not statistically different (cat: 22.2, grass: 24.4, HDM: 23.5, horse: 23.1, and ragweed: 23.0; ANOVA p = 0.824) (Table 3).

**Table 3:** Mean FEV_1_ and AUC (± SD) data and statistical analyses for common allergens

	Cat	Grass	HDM	Horse	Ragweed	ANOVA p-value	Significant pairwise comparisons
*EAR*_*(0-3 h)*_* FEV*_*1, max*_	30.0 ± 6.7	31.5 ± 7.8	35.5 ± 8.4	29.7 ± 7.9	36.9 ± 9.7	< 0.0001	Cat vs HDM, Cat vs Ragweed
*EAR*_*(0-3 h)*_* FEV*_*1, min*_	1.1 ± 7.8	2.5 ± 4.9	3.8 ± 5.2	1.7 ± 6.1	6.6 ± 5.6	0.002	Cat vs Ragweed
*EAR*_*(0-3 h)*_* Absolute* *Difference*	28.9 ± 7.8	28.9 ± 7.3	31.8 ± 8.2	28.0 ± 6.7	30.3 ± 10.4	0.180	N/A
*LAR*_*(3-7 h)*_* FEV*_*1, max*_	22.2 ± 6.8	24.4 ± 7.8	23.5 ± 7.1	23.1 ± 7.2	23.0 ± 7.0	0.824	N/A
*EAR*_*(0-3 h)*_* AUC (%FEV*_*1*_* x min)*	1917.0 ± 1091.4	2264.0 ± 1083.5	2510.2 ± 997.5	2089.9 ± 1178.8	3120.8 ± 1257.4	< 0.0001	Cat vs HDM, Cat vs Ragweed, Horse vs Ragweed
*LAR*_*(3-7 h)*_* AUC (%FEV*_*1*_* x min)*	3003.5 ± 1329.0	3697.0 ± 1638.8	3164.9 ± 1349.2	3325.4 ± 1993.2	3238.9 ± 1807.2	0.548	N/A

## Discussion

The correlation of FEV_1, max_ to AUC for all allergens combined during the EAR_(0-2 h)_ and LAR_(3-7 h)_ were both strong and did not differ statistically (r = 0.807 and 0.798 respectively; difference p = 0.408). Thus, a large FEV_1, max_ correlates to a large AUC for both the EAR_(0-2 h)_ and LAR_(3-7 h)_. This result is useful for future ABP tests, since it establishes a strong relationship between FEV_1, max_ and AUC for both the EAR_(0-2 h)_ and LAR_(3-7 h)_ in the context of all the allergens examined. Thus, a greater degree of bronchoconstriction, as measured by the FEV_1, max_, will likely result in a longer recovery period, as seen by the AUC. Future pharmacological studies aimed at measuring the recovery after an allergen-induced asthmatic response might pick either measure to demonstrate recovery, or choose a specific measurement based on the correlations outlined for the chosen allergen.

The LAR_(3-7 h)_ occurs over a longer time period than the EAR_(0-2 h)_, which we believed would allow for greater variability in AUC, thereby reducing the correlation between FEV_1, max_ and AUC; since these two correlation values did not differ, the AUC method can provide insight on the magnitude of response even during longer response periods. A previous study also found that the correlation between FEV_1_ and the area under the expiratory flow-volume curve in a methacholine challenge was strong (r = 0.939) [13]. Some key differences from that study are that the expiratory flow-volume curve and AUC are not direct substitutes for each other, and methacholine and ABP challenges do not cause bronchoconstriction through the same pathway with substantially different time courses; methacholine-induced bronchoconstriction is more rapid in both onset and recovery. Methacholine is a direct bronchoconstrictor, it binds to receptors on airway smooth muscle, while an ABP challenge is an indirect test that leads to bronchoconstriction through inflammatory mediators via the IgE pathway [2]. Nonetheless, our findings are in accordance with showing FEV_1_ and area under a curve describing air flow should correlate.

Pairwise comparisons of individual allergens’ correlation of FEV_1, max_ vs AUC showed grass (r = 0.935) had a statistically significant higher value compared to cat, HDM, horse, and ragweed during the EAR_(0-2 h)_ (p values range from < 0.0001 to 0.042). The between-participant variability of FEV_1, max_ and AUC for grass allergen tended to be less than that for other allergens. The allergen with the second highest correlation during the EAR_(0-2 h)_ was ragweed (r = 0.839), although this value only differed statistically to cat and grass (p = 0.035 and 0.042, respectively). Both grass and ragweed are seasonal allergens. ABP testing in these individuals was performed outside allergy season to avoid the potential for increased allergen responsiveness resulting from recent exposure. This is in contrast to perennial allergens like HDM, wherein exposure is chronic, potentially leading to enhanced responsiveness to ABP testing [6]. HDM had a lower correlation coefficient (r = 0.778) but only differed statistically to grass (p = 0.001). Perhaps the difference in correlation is due to the type of exposure: chronic exposure to an allergen leading to more responsive airways maybe associated with more between-participant variability, leading to a lower AUC vs FEV_1, max_ correlation. Nonetheless, cat allergen, had the lowest correlation (r = 0.650), and would only be a perennial allergen if the participant lived with a cat, but this value only differed statistically to grass. The choice between using FEV_1, max_ and/or AUC to demonstrate recovery is also dependent on the individual allergen being studied. The lower correlation for the cat allergen can be a guide for future ABP tests. It may be more useful to calculate both the FEV_1, max_ and AUC for the EAR_(0-2 h)_ of cat allergen compared to other allergens, since a large FEV_1, max_ does not have as strong of a correlation to a large AUC, and vice versa. Overall, only grass, a seasonal allergen, had statistically significant higher correlation than every other allergen tested.

Correlation and slope values of FEV_1, max_ vs AUC during the LAR_(3-7 h)_ did not show a significant difference between most allergens, only the HDM vs ragweed slope comparison was statistically significant (slope = 140.2 and 216.2 respectively; p = 0.034). The slope of the regression lines for AUC vs FEV_1, max_ for all allergens during the LAR_(3-7 h)_ was steeper than that of the EAR_(0-2 h)_ (slope = 161.1 and 81.9 respectively; p < 0.0001). During the EAR_(0-2 h)_, the only significant pairwise comparisons for the slope of FEV_1, max_ vs AUC was cat vs grass (slope = 70.6 and 102.9 respectively; p = 0.013) and grass vs HDM (slope = 102.9 and 71.7 respectively; p = 0.002). The EAR_(0-2 h)_ slope is related to the recovery period following allergen inhalation (once FEV_1, max_ is reached, FEV_1_ would approach baseline and AUC would decrease), whereas the LAR_(3-7 h)_ slope is a function of the magnitude of the LAR_(3-7 h)_ (i.e., the development of the response as a sustained drop in FEV_1_ which would result in a large AUC). No one allergen resulted in a difference in recovery after allergen inhalation (i.e., EAR_(0-2 h)_), or in the magnitude of the LAR_(3-7 h)_, than all other allergens. Specifically, since HDM did not have significantly different slopes, we cannot conclude that HDM caused a longer recovery period, or a larger LAR_(3-7 h)_ magnitude. The slope values may also be influenced by outliers, especially at larger FEV_1, max_ values (≥ 45) where the points were much more dispersed.

Based on Fig. 1, it is possible that grass is the only allergen undergoing recovery during the 6-to-7-h period during the LAR_(3-7 h)_. All other allergens appear to still be increasing or reaching their maximum FEV_1_ at 7 h, but we would need to have data past this time point, until the maximum response is reached, to be able to comment on LAR_(3-7 h)_ recovery. During the EAR_(0-3 h)_, ragweed had both the largest FEV_1, max_ and AUC, followed by HDM. However, these values are not significantly different compared to the other allergens. During the LAR_(3-7 h)_ the allergen with the largest FEV_1, max_ and AUC were not the same: HDM had the largest FEV_1, max_ while grass had the largest AUC. Importantly though, neither the LAR_(3-7 h)_ FEV_1, max_ nor the AUC values differed statistically between allergens.

The absolute difference in the highest and lowest percent fall in FEV_1_ during the EAR_(0-3 h)_ did not differ between allergens (p = 0.180), while the EAR_(0-3 h)_ AUC did (p < 0.0001). However, HDM did not result in a larger AUC than all the other allergens; the only significantly different pairwise comparisons were cat vs HDM, cat vs ragweed, and horse vs ragweed. No single allergen had a statistically larger EAR_(0-3 h)_ AUC than the rest. Thus, we cannot conclude from these findings that the recovery after the EAR_(0-3 h)_ for HDM allergen is longer than other allergens, the same conclusion we reached when comparing EAR_(0-2 h)_ FEV_1, max_ vs AUC slopes. We suspected that HDM would result in a delayed or slower recovery during the EAR_(0-3 h)_ because of previous research showing more severe ABP results [6, 7], as well as the activation of additional proteolytic pathways that other allergens may not induce [9–11]. Our findings may not be in accordance with previous data due to ragweed having the largest EAR_(0-3 h)_ AUC, followed by HDM (3120.8 and 2510.2, respectively) with ragweed having a much smaller sample size compared to HDM (n = 26 and 72, respectively). Perhaps the smaller group of participants challenged with ragweed had more severe responses and thus larger EAR_(0-3 h)_ AUCs than average. Despite the smaller sample size of the ragweed group, HDM still did not cause a larger AUC than all other allergens. The additional mechanisms previously described to account for HDM-induced bronchoconstriction may still be occurring, we simply did not find evidence to suggest this bronchoconstriction or recovery thereafter was more severe or prolonged.

A limitation of our research is the small sample size of some allergen groups, preventing us from analysing them as individual allergens. For example, *Alternaria* and tree had 5 (all had LARs) and 2 (only one dual responder) participants. These participants were included in the combined allergen correlation analyses for both the EAR and LAR, but *Alternaria* and tree could not be compared as individual allergens to the other groups (cat, grass, HDM, horse, and ragweed). Some differences in the data may be due to the small and variable group sizes rather than true differences. Nonetheless, the Scheffe test is considered a more conservative *post-hoc* test [12]. Additionally, the missing time points for three participants meant we had to interpolate these percent fall in FEV_1_ values using a weighted mean. However, this is unlikely to influence the overall trend of the data when we analyzed all 221 participants.

Overall, the correlation between AUC vs FEV_1, max_ is strong and did not differ during the EAR_(0-2 h)_ and LAR_(3-7 h)_ (r = 0.807 and 0.798 respectively; difference p = 0.408). This result allows us to better understand the endpoints used to measure ABP tests. Participants with severe bronchoconstriction, as seen by a large percent fall in FEV_1_, will also likely have a large magnitude of response, as seen by a large AUC during both the EAR_(0-2 h)_ and LAR_(3-7 h)_. Although we predicted HDM to cause a slower recovery and thus have a larger EAR_(0-3 h)_ AUC due to possible additional proteolytic pathways and perennial exposure, we did not find evidence to support this claim [6, 9–11]. The EAR_(0-3 h)_ and LAR_(3-7 h)_ AUC as well as the EAR_(0-3 h)_ and LAR_(3-7 h)_ FEV_1, max_ for HDM was not larger than all the other allergens tested.

Future research could be done to understand if the correlation between FEV_1, max_ and AUC also exists in challenges outside of ABP testing, such as direct acting stimuli like methacholine. In addition, the recovery after an HDM challenge could be measured in terms of the absolute change in in FEV_1, max_ to FEV_1, min_ (L) rather than percent fall in FEV_1_. Using liters rather than percent fall from baseline would control for allergens that cause a larger fall in percent FEV_1_ and thus their recovery can be smaller to meet the same absolute difference as other allergens.

## Data Availability

The datasets used and/or analysed during the current study are available from the corresponding author on reasonable request.

## Abbreviations

ABP: Allergen bronchoprovocation
FEV_1_: Forced expiratory volume in one second
AUC: Area under the FEV_1_ vs time curve
HDM: House dust mite
EAR: Early asthmatic response
LAR: Late asthmatic response
FEV_1, max_: Maximal percent fall in FEV_1_
IgE: Immunoglobulin-E
FEV_1, min_: Minimal percent fall in FEV_1_
SD: Standard deviation
